# Influence of Increase in Phosphorus Supply on Agronomic, Phenological, and Physiological Performance of Two Common Bean Breeding Lines Grown in Acidic Soil under High Temperature Stress Conditions

**DOI:** 10.3390/plants12183277

**Published:** 2023-09-15

**Authors:** Juan Carlos Suárez, Milan O. Urban, José Alexander Anzola, Amara Tatiana Contreras, José Iván Vanegas, Stephen E. Beebe, Idupulapati M. Rao

**Affiliations:** 1 Programa de Ingeniería Agroecológica, Facultad de Ingeniería, Universidad de la Amazonia, Florencia 180001, Colombia; ing.agroec.anzola@gmail.com (J.A.A.); amaratatis18@gmail.com (A.T.C.); jos.vanegas@udla.edu.co (J.I.V.); 2 Centro de Investigaciones Amazónicas CIMAZ Macagual César Augusto Estrada González, Grupo de Investigaciones Agroecosistemas y Conservación en Bosques Amazónicos-GAIA, Florencia 180001, Colombia; 3 International Center for Tropical Agriculture (CIAT), Km 17 Recta Cali-Palmira, Cali 763537, Colombia; m.urban@cgiar.org (M.O.U.); s.beebe@cgiar.org (S.E.B.); i.rao@cgiar.org (I.M.R.); 4 Programa de Maestría en Sistemas Sostenibles de Producción, Facultad de Ingeniería, Universidad de la Amazonia, Florencia 180001, Colombia

**Keywords:** heat stress, grain yield, energy use, chlorophyll fluorescence, photosynthate remobilization

## Abstract

Many common bean (*Phaseolus vulgaris* L.) plants cultivated in areas of the world with acidic soils exhibit difficulties adapting to low phosphorus (P) availability, along with aluminum (Al) toxicity, causing yield loss. The objective of this study was to evaluate the influence of an increase in P supply level on the agronomic, phenological, and physiological performance of two common bean breeding lines grown in acidic soil, with low fertility and under high temperature conditions, in a screenhouse. A randomized complete block (RCB) design was used under a factorial arrangement (five levels of P × 2 genotypes) for a total of 10 treatments with four replications. The factors considered in the experiment were: (i) five P supply levels (kg ha^−1^): four levels of P0, P15, P30, and P45 through the application of rock phosphate (RP), and one P level supplied through the application of organic matter (PSOM) corresponding to 25 kg P ha^−1^ (P25); and (ii) two advanced bean lines (BFS 10 and SEF10). Both bean lines were grown under the combined stress conditions of high temperatures (day and night maximum temperatures of 42.5 °C/31.1 °C, respectively) and acidic soil. By increasing the supply of P, a significant effect was found, indicating an increase in the growth and development of different vegetative organs, as well as physiological efficiency in photosynthesis and photosynthate remobilization, which resulted in higher grain yield in both bean lines evaluated (BFS 10 and SEF10). The adaptive responses of the two bean lines were found to be related to phenological adjustments (days to flowering and physiological maturity; stomatal development), as well as to heat dissipation strategies in the form of heat (NPQ) or unregulated energy (qN) that contributed to greater agronomic performance. We found that, to some extent, increased P supply alleviated the negative effects of high temperature on the growth and development of the reproductive organs of bean lines. Both bean lines (BFS 10 and SEF 10) showed adaptive attributes suited to the combined stress conditions of high temperature and acidic soil, and these two lines can serve as useful parents in a bean breeding program to develop multiple stress tolerant cultivars.

## 1. Introduction

It is estimated that about 60% of cultivated areas of common bean (*Phaseolus vulgaris* L.) have low soil fertility problems associated mainly with nutrient deficiencies [1,2] as well as the presence of aluminum (Al) toxicity in acid soils [3]. The low fertility of soils in the tropics is mainly due to the deficiency of some essential elements—such as phosphorus (P) [4]—that are important for plant growth; this causes major limitations for shoot and root growth [5,6]. Specifically, aluminum sensitivity is localized in the root apex, which reduces total root length, surface area, and secondary root branching patterns that impacts root elongation and nutrient uptake [7]. In addition to low soil fertility, farmers are faced with temperature increases [8,9] that can cause heat waves [10,11] which could lead to a 50% loss of the planted area by 2050, according to different predictions regarding an expected increase in heat stress [12,13,14].

The common bean is the most important grain legume in the human diet [15,16], and it is a nutrient-demanding crop that can cope with varied environmental stress conditions [17]. It is more sensitive to high temperatures than the other grain legumes [12,18], a situation that requires improved understanding of the specific adaptation mechanisms of bean lines for achieving heat tolerance in bred lines [12,19]. Based on several studies conducted in different environments [20,21,22,23], heat stress in bean plants generates a series of irreversible injuries to plant metabolism and development [20,21,23,24,25]. These changes often result in decreased bean productivity due to a reduction in carbon assimilation and biomass accumulation [10,23,26]; affecting flower production, pollen viability, and pod formation; and decreasing the seed number, seed size, and seed yield [21,23,27]. Similarly, at higher temperatures (35–42 °C), several physiological processes are affected, disrupting the biochemical reactions of photosynthesis, including the functioning of chloroplasts and enzymes [28]. These changes induce stomatal closure that limits CO_2_ diffusion from the stomata through intercellular spaces and carboxylation sites throughout the leaf mesophyll cells [29]. However, some plants develop several adaptive mechanisms to cope with heat stress [30], such as the production of heat shock proteins, to protect cellular components from damage caused by high temperatures [28,31].

In grain legumes such as beans, P, as an essential nutrient, plays a central role in energy conservation and transfer in cellular metabolism [32], as well as in plant growth, development, and yield [2,33]. The P availability in soil is one of the main constraints for bean production in the tropics [1,2,34], and if the amount of P available in the soils does not meet the requirements of the beans, certain mismatches will be generated, altering the partitioning of P between plant parts [35]. In response to P deficiency, plants develop adaptive mechanisms at the morphological, physiological, and biochemical level [36,37,38,39] by mobilizing more P to the reproductive parts at the expense of the P concentration available to the vegetative parts [40,41].

Adequate P supply is necessary to enhance nodulation, symbiotic efficiency, and nutrient uptake in the common bean [42,43,44]. The supply of P is necessary for biomass accumulation, the translocation of assimilates (source-sink) [45], and the production of protein-containing compounds, mainly in seeds [46]. Adequate P supply has previously been shown to help offset the impact of drought stress on grain yield and quality [39,47], in addition to increasing water use efficiency and dry matter accumulation [48]. However, it previous studies have not determined whether an increase in P supply alleviates the stress caused by high temperatures in acidic soil regions.

In the Amazon region, bean lines have been evaluated for their adaptation to the combined stress conditions of low fertility acidic soils and high temperature in order to improve the diet of smallholder farmers and children living in rural areas, and a few promising lines were identified [22,23,24]. This study aimed to evaluate the influence of an increase in P supply on the agronomic, phenological, and physiological performance of two common bean breeding lines grown in acidic soil under high temperature stress conditions. The study was conducted in a screenhouse under natural conditions that induced the combined stress conditions of high temperature and acidic soil to the grown plants. These combined abiotic stress conditions are common in smallholder agriculture in the Amazon region. We tested the hypothesis that an increased P supply to acidic soil alleviates the effects of high temperature stress on the growth, development, and yield of two genetically adapted bean lines.

## 2. Results

### 2.1. Dynamics of Growth and Development of Different Vegetative Organs under Heat Stress

**Figure 1:**
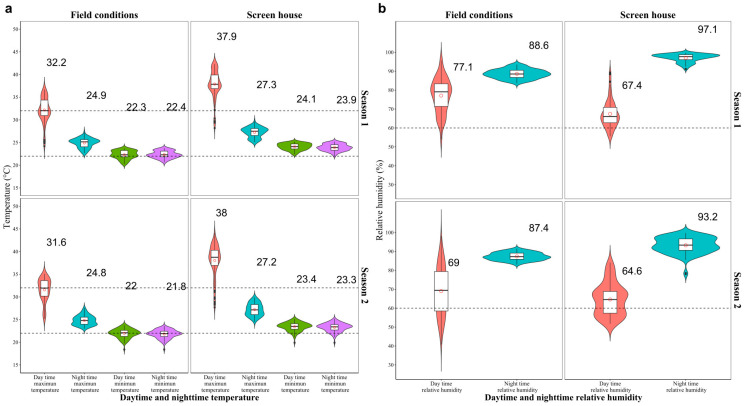
Distribution of maximum and minimum day and night temperatures and relative humidity during the growing season at the Macagual Research Center in Colombia over two seasons (2019) and (2020) under outside (field conditions) and inside (screenhouse) conditions. (**a**) Temperature—the dotted horizontal line denotes 22 °C and 32 °C; (**b**) relative humidity (%)—the dotted horizontal line denotes 60% relative humidity.

During the evaluation time of the study on the effects of different P supply levels, the mean daily temperature was 28.2 ± 0.2 °C, while the minimum and maximum values were 21.7 and 42.5 °C, respectively. By analyzing the differences during the day and night in relation to the minimum and maximum temperature, we found that during the day, the mean minimum and maximum temperature values were 23.9 ± 0.1 and 37.1 ± 0.2 °C, respectively. During the night, the mean minimum and maximum temperature values were 23.7 ± 0.1 °C and 28.1 ± 0.1 °C, respectively (*p* < 0.05, Figure 1). The temperatures under field conditions (outside the screenhouse) were presented to show the extent of the temperature increase in the screenhouse during both the day and night. 

**Figure 2:**
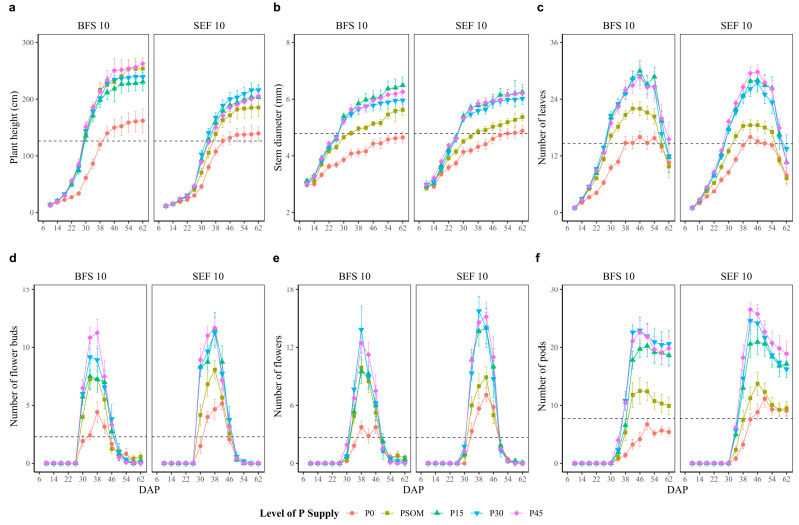
Response of growth and development of different vegetative organs of two bean lines (BFS 10, SEF 10) at different sampling times (days after planting, DAP) as a function of different levels of P supply (P0, P15, P30, and P45 kg ha^−1^; and P supplied through organic matter (PSOM)) to acidic soil under high temperature stress conditions. (**a**) Plant height (cm); (**b**) stem diameter (mm); (**c**) number of leaves; (**d**) number of flower buds; (**e**) number of flowers; (**f**) number of pods. The dotted black line corresponds to the average of each variable. The results include mean ± SE (n = 20).

Under this heat stress condition, the bean lines exhibited contrasting responses to the increased level of P supply in terms of the growth dynamics and the development of different vegetative organs (*p* < 0.05). In general, heat stress affected the growth and development dynamics of the different bean lines when the P supply was zero (Figure 2, red line treatment), in contrast to the results for the other P treatments. By increasing the P supply, the bean lines showed a tendency to dissipate the heat stress, which resulted in an increase in the growth rate and the development of different plant organs. When we compared the variables between treatments, we found that the control treatment (P0) presented an average plant height value of 120 cm, much lower than the results for treatments P30 and P45, with plant height values of 201 and 213 cm, respectively (Figure 2a). When comparing the plant height between bean lines, in all treatments evaluated, the BFS 10 plants were taller than the SEF 10 samples. A similar trend was observed for stem diameter (Figure 2b). In regards to the number of leaves, for the two bean lines, the maximum number of leaves per plant was 29, which was reached at 46 DAP at the P45 supply level, compared to the 15 leaves obtained for the P0 (control) treatment (Figure 2c). 

It was found that the higher temperature stress in the screenhouse significantly affected flower bud formation, and this effect was more evident at the P0 level (Figure 2d, red line treatment) compared to the results observed for other treatments. Heat stress affected the phenological process, since at 30 DAP, both lines presented flower buds, but with greater number in the treatments with increased P supply for SEF 10 (Figure 2d). In the case of flowers, at 34 DAP, the first difference is noted, with a maximum value at 38 DAP, with 15 and 13 flowers for SEF 10 and BFS 10, respectively (Figure 2e). In the end, the number of pods formed was affected by an increase in temperature above 22 °C (Figure 2f). With the control treatment (P0), the maximum number of pods formed was 11 and 6 for SEF 10 and BFS 10, respectively. However, increasing the P supply increased the number of pods formed, reaching a maximum of 26 and 22 for SEF 10 and BFS 10, respectively, at a P supply level of P45 (Figure 2f).

**Figure 3:**
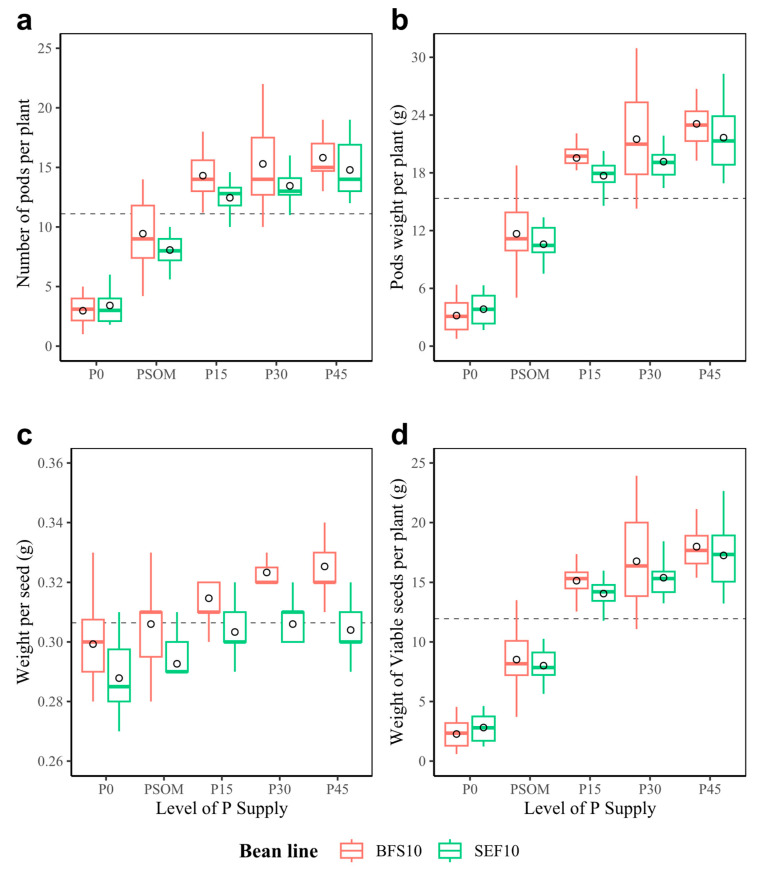
Pod formation and seed weight of two bean lines (BFS 10, SEF 10) evaluated using different levels of P supply (P0, P15, P30, and P45 kg ha^−1^; and P supplied through organic matter (PSOM)) to acidic soil under high temperature stress conditions. (**a**) Number of pods per plant; (**b**) pod weight per plant (g); (**c**) weight per seed (g); (**d**) seed weight per plant (g). The dotted black line corresponds to the average of each variable. The circle in each box means the average. The results include mean ± SE (n = 20).

As for the variables related to pod and seed formation, under high temperature conditions, increasing the P supply significantly increased the number of pods and seeds in both bean lines evaluated (*p* < 0.05, Figure 3). For example, the average number of pods per plant increased from 3 to 15 for the P0 and P45 treatments, respectively (Figure 3a). A similar situation was found for pod weight per plant, in which production reached an average of 4 g in P0 to more than 18 g per plant when the P supply was increased from 15 kg ha^−1^ (P15, Figure 3b). Between bean lines, differences in individual seed weight were found, with the difference being more evident in the P45 treatment (Figure 3c). In the end, it was found that the P supply significantly increased the number of pods per plant, as well as the seed weight, which resulted in an increase in the seed yield per plant (Figure 3d).

**Figure 4:**
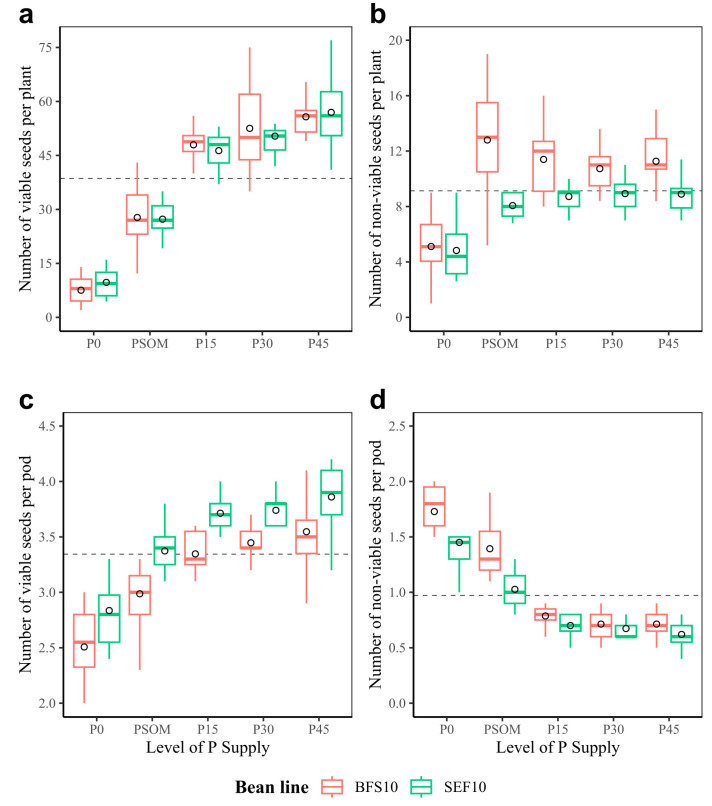
Seed viability of two bean lines (BFS 10, SEF 10) evaluated using different levels of P supply (P0, P15, P30, and P45 kg ha^−1^; and P supplied through organic matter (PSOM)) to acidic soil under high temperature stress conditions. (**a**) Number of viable seeds per plant; (**b**) number of non-viable seeds per plant; (**c**) number of viable seeds per pod; (**d**) number of non-viable seeds per pod. The dotted black line corresponds to the average of each variable. The circle in each box means the average. The results include mean ± SE (n = 20).

Under the screenhouse conditions in which the experiment was carried out, it was found that the level of viability of seeds per plant increased with the increasing supply of P (Figure 4a), a situation that also occurred in the non-viable seeds. This is due to the relationship between the increasing P supply and the production of more seeds per plant (Figure 4b). The above behavior was also observed in regards to the number of viable (Figure 4c) and non-viable seeds per pod (Figure 4d), where increasing the supply of P also increased the viability of the seeds.

### 2.2. Phenological Characteristics, Stomatal Density, and Dynamics of Morphological Development

When analyzing the effect of high temperature combined with P supply on the phenological responses of the genotypes evaluated (BFS 10 and SEF 10), we found significant differences between treatments and genotypes (*p* < 0.01). We observed that the increase in temperature decreased the number of days to flowering for both bean lines, with earlier flowering under all treatments. We also noted that the plants reached the flowering stage in a shorter period of time (30 DAP) with an increased P supply (P15, P30, P45) (Supplementary Figure S2a). A similar trend was observed regarding the days to physiological maturity, with maturity reached at 58 and 60 days for SEF 10 and BFS 10, respectively, under increasing P treatments (Supplementary Figure S2b). The pollen viability (PV) values were lower with P0 treatment under heat stress (Supplementary Figure S2c). As the P supply level increased, the PV values presented contrasting behaviors. The BFS 10 line showed its highest PV value under the P30 treatment (>80%), and for SEF 10, the increase in its PV value corresponded to the increase in P supply treatments, reaching a value of up to 85% for the P45 treatment (Supplementary Figure S2c).

Stomatal density (SD) in both leaves and pods was inversely proportional to the increase in P supply to the soil. We found that with P0 treatment, BFS 10 presented a stomatal density of 73 mm^−2^ in the leaves, a decrease of 13% compared with that recorded for the P45 treatment (63 mm^−2^) (Supplementary Figure S2d). In the case of SD in the pods, this value was found in a lower proportion than that in the leaves, and we observed that under the PSOM (53 mm^−2^), P15 (49 mm^−2^), and P45 (47 mm^−2^) treatments, an increase in SD value was observed compared to that noted for the P0 (32 mm^−2^) and P30 (38 mm^−2^) treatments. The SEF 10 line showed a higher SD value than that of the BFS 10 line, under all P treatments. The SD value of SEF 10 also decreased with the increase in P supply, presenting an SD value of 75 mm^−2^ with the P0 treatment and showing a decrease of 18% with the P45 treatment (61 mm^−2^). The SD value in the SEF 10 pods was similar, with no differences observed among P treatments, showing an average value of 37 mm^−2^ (Supplementary Figure S2d).

The specific leaf area (SLA) parameter showed a significant effect in the genotype × treatment interaction for both growth stages (flowering and pod filling) (*p* < 0.01, Supplementary Figure S3a). The SLA value for BFS 10 at the flowering stage (52 m^2^ kg^−1^) and at the pod filling stage (60 m^2^ kg^−1^) was higher under the P0 treatment compared to the results for the other P supply treatments, where it increased with increasing P supply, in contrast to what was observed with SEF 10, in which the SLA value was higher under PSOM treatment (56 m^2^ kg^−1^). The leaf thickness showed variations among P treatments, as well as between the two bean lines evaluated. For example, SEF 10 showed a greater value of leaf thickness compared to that of BFS 10. Among P treatments, under the P45 treatment, the maximum value observed was 0.75 mm at the flowering stage (Supplementary Figure S3b). 

Leaf growth showed similar trends for both bean lines at different stages of vegetative development. From the second sampling time onwards, there were differences between the treatments using an increased P supply compared to the P0 treatment (Supplementary Figure S4). We observed that the leaf development of both lines differed, with an increase in leaf width for SEF 10 and a greater leaf length for BFS 10 (Supplementary Figure S4a). At the V_3_ stage (1st trifoliate leaf), an increase in leaf expansion (higher SLA values) was more noticeable in the treatments with increased P supply, specifically with the P45 treatment. These increases in P supply favored leaf growth, i.e., the leaf length was 54 and 72 mm for BFS 10 and SEF 10, respectively (Supplementary Figure S4a), and the leaf width was 30 and 38 mm for these same lines, respectively (Supplementary Figure S4b), compared to the 13 and 33 mm leaf width and leaf length, respectively, noted for the P0 treatment. This leaf growth behavior in both the pre-flowering and flowering stages was more significant under the increased P supply treatments (Supplementary Figure S4). 

The high temperature negatively affected growth in terms of pod length and width, a situation that was markedly alleviated by increasing the supply of P in the soil. When we analyzed the increase in growth in both the length and width of the pod, the highest values occurred between the 1st and 3rd sampling times, and after these two sampling times, the slope of growth was reduced in the two bean lines evaluated. Between the two bean lines, we found, in the second sampling time specifically, significant differences between P treatments for BFS 10 compared to SEF 10 (Supplementary Figure S5a). When comparing P treatments independent of bean lines, we found contrasting differences in pod lengths between the P0 and P45 treatments, with values of 85 and 106 mm, respectively. In the case of pod width, BFS 10 presented higher values compared to those of SEF 10 (Supplementary Figure S5b).

### 2.3. Efficiency in Assimilate Mobilization: Canopy Biomass, Dry Matter Partitioning, Yield, and Yield Components

**Figure 5:**
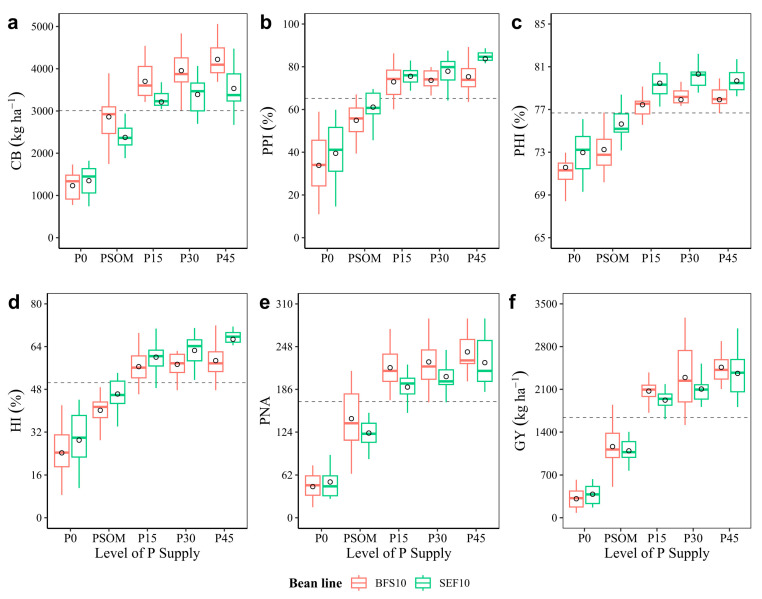
Dry matter production, dry matter partitioning, and grain yield response of two bean lines (BFS 10, SEF 10) to different levels of P supply (P0, P15, P30, and P45 kg ha^−1^; and P supplied through organic matter (PSOM)) to acidic soil under high temperature stress conditions. (**a**) Canopy biomass (CB); (**b**) pod partitioning index (PPI); (**c**) pod harvest index (PHI); (**d**) harvest index (HI); (**e**) pod number per area (PNA); and (**f**) grain yield (GY). The dotted black line corresponds to the average of each variable. The circle in each box means the average. The results include mean ± SE (n = 20).

Heat stress caused a marked reduction in canopy biomass (CB) production with P0 treatment (1230 kg ha^−1^) compared to P45 treatment (4221 kg ha^−1^), and this response to the increase in P supply in regards to CB values was very similar for the two lines evaluated (Figure 5a). Regarding the ability to mobilize photosynthates for pod formation, based on dry matter partitioning indices, the high temperature decreased the pod partitioning index (PPI) values by 40% in both lines, when compared with the control treatment (P0). This situation changed markedly when the P supply was increased, in which case, the biomass partitioning for pod formation was higher than 70% (Figure 5b). When analyzing the mobilization of assimilates from the pod wall to seed formation (pod harvest index, PHI), a similar trend was observed for PPI (Figure 5c). At physiological maturity, the harvest index (HI) value was above 50% when the P supply was increased, and this is contrary to what was observed with the control treatment (P0), in which the HI value was around 25% for both bean lines evaluated (Figure 5d). As for the pod number per area (PNA), the increase in PNA was proportional to the increase in grain yield (GY), with BFS 10 being higher at 240 pods m^−2^ and SEF 10 lower, with 225 pods m^−2^ under the P45 treatment (Figure 5e). In the case of grain yield, the response of the two bean lines under high temperature conditions under the P0 treatment was very low (kg ha^−1^: BFS 10 = 311; SEF 10 = 385) compared to that of the increased P supply treatments (kg ha^−1^: P45 = 2460 and 2361 kg ha^−1^, for BFS 10 and SEF 10, respectively Figure 5f).

### 2.4. Chl_a_ Fluorescence and Images of Chlorophyll (Chl_a_) Parameters under Different Levels of P Supply

**Figure 6:**
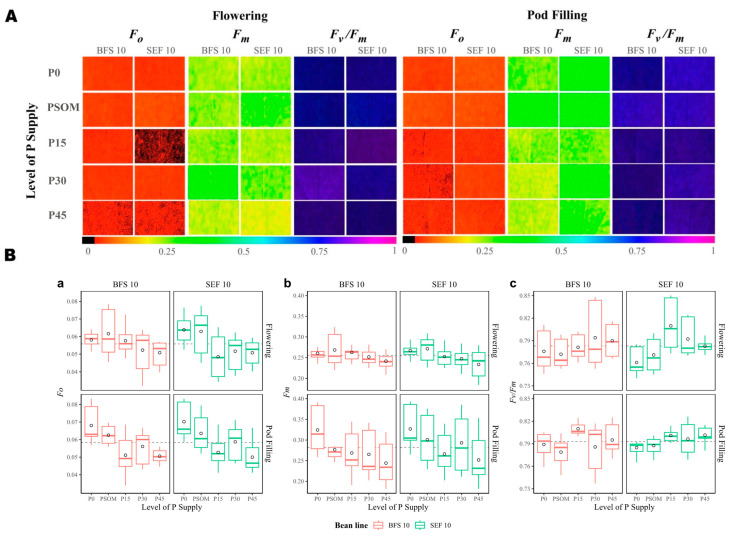
Photosynthetic performance of two bean lines (BFS 10, SEF 10) in response to different levels of P supply (P0, P15, P30, and P45 kg ha^−1^; and P supplied through organic matter (PSOM)) to acidic soil under high temperature stress conditions. (**A**) Images of chlorophyll a fluorescence parameters. The change in the color from red to violet shows an increase from lower to higher values. Images were taken from fully developed leaves at the onset of physiological maturity: initial fluorescence (F_0_); maximum fluorescence (F_m_); maximum quantum efficiency of PSII (F_v_/F_m_). (**B**) The phosphorus supply induced changes in the chlorophyll *a* fluorescence parameters. (a) Initial fluorescence (F_0_); (b) maximum fluorescence (F_m_); (c) maximum quantum efficiency of PSII (F_v_/F_m_). The circle in each box means the average. The results include mean ± SE (n = 20).

According to the images of Chl_a_ fluorescence parameters and the quantitative analysis, the magnitude of the changes in F_0_ was proportional to the increase in P level, with the changes under the P45 treatment at the flowering stage for both BFS 10 and SEF 10 being more evident (Figure 6A). However, for F_m_ in BFS 10, a behavior inversely proportional to the P increase was noted at both the flowering and pod filling growth stages (Figure 6A). SEF 10 presented a higher magnitude of F_m_ value with PSOM treatment at the flowering stage, while at the pod filling stage, the decrease in F_m_ value was proportional to the increase in P supply (Figure 6B). The maximum efficiency of the PSII photochemistry (F_v_/F_m_) values ranged between of 0.74 and 0.85 (Figure 6A), with the values being lower under the P0 treatment at the two growth stages evaluated. For both BFS 10 and SEF 10, the F_v_/F_m_ values were higher with an increasing supply of P. 

**Figure 7:**
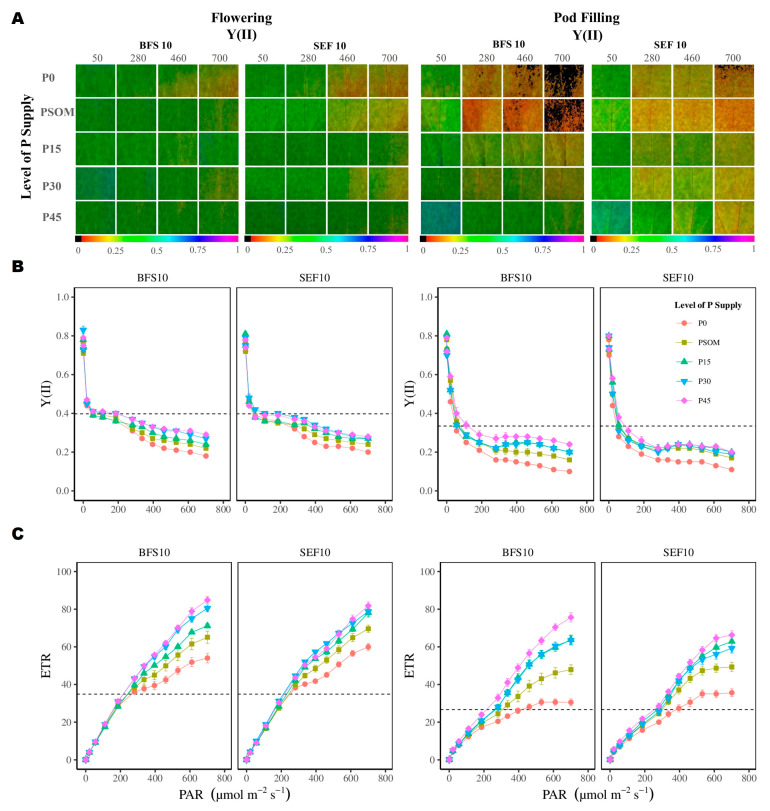
Photosynthetic performance of two bean lines (BFS 10, SEF 10) at the flowering and pod filling growth stages in response to different levels of phosphorus supply (P0, P15, P30, and P45 kg ha^−1^; and P supplied through organic matter (PSOM)) to acidic soil under high temperature stress conditions. (**A**) Images of chlorophyll a fluorescence parameters. The change in the color from red to violet shows an increase from lower to higher values. Images were taken from fully developed leaves at the onset of physiological maturity. (**B**) The phosphorus supply induced changes in Y(II) (yield of photochemistry) in relation to the increase in photosynthetically active radiation (PAR). (**C**) The phosphorus supply induced changes in ETR (electron transport rate) in relation to the increase in PAR.

In general, the Y(II) (yield of photochemistry) was inversely proportional to the increase in PAR, the value being different at both growth stages, as well as under increased P supply levels for both BFS 10 and SEF 10. Comparing among the P treatments, the P0 treatment presented the lowest Y(II) values (Figure 7B). The electron transport rate (ETR) increased with increasing photosynthetically active radiation (PAR), the behavior between the two different growth stages being significantly different. When compared between the two bean lines, the ETR value was higher for BFS 10 than for SEF 10 (Figure 7C).

**Figure 8:**
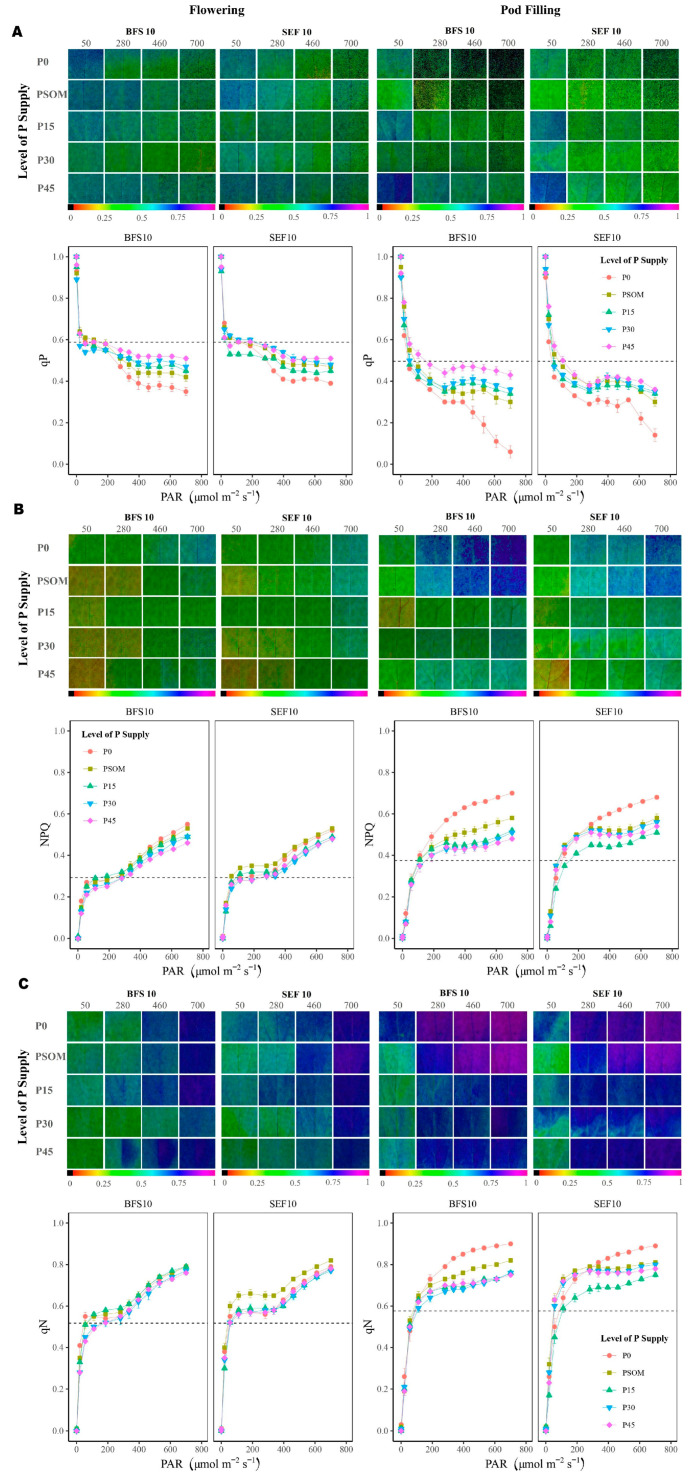
Images of chlorophyll *a* fluorescence parameters of two bean lines (BFS 10, SEF 10) in response to different levels of P supply (P0, P15, P30, and P45 kg ha^−1^; and P supplied through organic matter (PSOM)) to acidic soil under high temperature stress conditions. The change in the color from red to violet shows an increase from lower to higher values. The images were taken from fully developed leaves at the onset of physiological maturity. (**A**) Phosphorus supply induced changes in the yield of photochemistry (qP) in relation to the increase in photosynthetically active radiation (PAR); (**B**) the phosphorus supply induced changes in the yield of dissipation by downregulation (NPQ) in relation to the increase in photosynthetically active radiation (PAR); (**C**) the phosphorus supply induced changes in the yield for other non-photochemical (non-regulated) losses (qN) in relation to the increase in photosynthetically active radiation (PAR).

When analyzing the different fractions of the routes that energy can take in the photosynthesis process, it was found that the qP (photochemical route) was higher compared to dissipation in the form of heat (NPQ) and the unregulated (qN) energy pathway (Figure 8). The values of qP were higher at the flowering stage, and these values increased with an increase in P supply, with no differences between the two bean lines evaluated (Figure 8A). However, at the pod formation stage, the fraction of qP was lower compared to that presented at flowering, with additional variations observed with different P supply treatments. Among the P treatments and the two bean lines evaluated for qP during the pod filling period, BFS 10 showed higher a value under P45 treatment (Figure 8A).

For energy dissipated as heat (NPQ), the same trend was generally observed with the increased P supply treatments for the two bean lines evaluated. Based on the color gradient in the image panel in Figure 8B, during the pod filling growth stage, we observed that it tended to be blue, with the highest NPQ value achieved with the P0 treatment for BFS 10, contrary to that presented for SEF 10. For qN (Figure 8C), which is the unregulated energy pathway, the value increased from a PAR level of 600 μmol m^−2^ s^−1^ at the flowering stage. However, this behavior changed considerably during the pod filling growth stage, in which the qN value increased from a PAR value of 200 μmol m^−1^ s^−1^.

**Figure 9:**
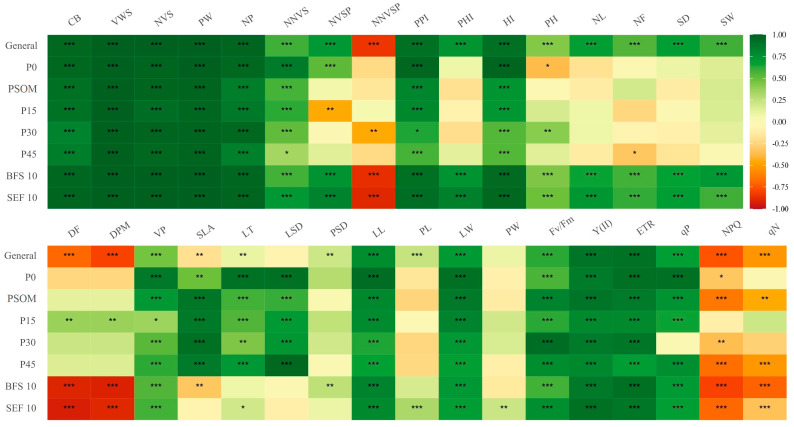
Correlations between grain yield (GY) and different agronomic, phenological, and physiological variables of two bean lines (BFS 10, SEF 10) evaluated with different levels of P supply (P0, P15, P30, and P45 kg ha^−1^; and P supplied through organic matter (PSOM)) to acidic soil under high temperature stress conditions. CB, canopy biomass; VWS, viable seed weight per plant; NVS, number of viable seeds per plant; PW, pod weight per plant; NP, number of pods per plant; NNVS, number of non-viable seeds per plant; NVSP, number of viable seeds per pod; NNVSP, number of non-viable seeds per pod; PPI, pod partitioning index; PHI, pod harvest index; HI, harvest index; PH, plant height; NL, number of leaves; NF, number of flowers; SD, stem diameter; SW, 100-seed weight; DF, days to flowering; DPM, days to physiological maturity; PV, pollen viability; SLA, specific leaf area; LT, leaf thickness; LSD, leaf stomatal density; PSD, pod stomatal density; LL, leaf length; PL, pod length; LW leaf width; PW, pod width; Fv/Fm, maximum quantum efficiency of PSII; Y(II), yield of photochemistry; ETR, electron transport rate; qP, yield of photochemistry; NPQ, yield for dissipation by downregulation; qN, yield for other non-photochemical (non-regulated) losses. *, ** and *** indicate significant correlations with 10%, 5% and 1% probability level, respectively.

When analyzing the relationship between GY and the different analyzed variables, we found that agronomic variables such as CB, WVS, NVS, WP, and NP, as well as physiological variables such as Y(II), ETR, and qP, showed a positive correlation greater than 0.75 (Figure 9). However, agronomic variables such as NNVSP, phenological variables such as DF and DPM, as well as some physiological variables such as NPQ and qN presented negative correlations with GY. When analyzing among factors, for example, BFS 10 presented correlations with traits at the leaf level (SLA and DSP), in contrast to SEF 10, which presented correlations between GY and leaf thickness (Th). Likewise, among the factors, only SEF 10 presented a positive correlation between GY and PL and PW. By increasing the P supply up to 45 kg ha^−1^, we found that the flower number increased, and this increase in flower number was positively correlated with an increase in GY (Figure 9).

## 3. Discussion

### 3.1. Increased Phosphorus Supply Alleviated, to Some Extent, the High Temperature Stress Effects on Growth and Development of Reproductive Organs 

In our study, under the higher temperature conditions, we observed a reduction in the growth rate and grain yield [21,23], specifically impacting the development of pods and seeds [18]. A mean night temperature higher than 25.9 °C reduced the viability and formation of flower buds, as the number of flowers produced was affected by heat stress [49,50], which subsequently impacted the formation of pods and the mobilization of assimilates for seed filling [23]. At the physiological level, heat stress is known to affect carbon assimilation, mainly during the reproductive phase [22], a situation that might have generated limitations in the loading of assimilates from the source to the sink, which could reduce pod development and grain yield [51]. 

We observed that the growth and development of both lines showed earlier evasion strategies to cope with heat stress. When comparing the behavior of the BFS 10 line with the responses obtained by Suárez et al. [23] under high temperature conditions, in our study, we found an enhancement of about 10 days in terms of earlier flowering and maturity under combined stress conditions compared to the earlier study in which the P supply was not limited. This allowed this line (BFS 10) to increase flower bud and pod production as a compensatory strategy to cope with abortion caused by heat stress. Previous research indicated that under a high temperature field conditions [23] and in soil with low fertility [21], the BFS 10 line reached an average pod production per area of 180 [21,22]. In the present study, we found that BFS 10 responded to an increase in P supply with an increase in both pod and seed production per plant (P45 = 240 pods). This is because the number of seeds per pod almost doubled, resulting in more than 50 seeds per plant under the P45 treatment compared to 10 seeds per plant under the P0 treatment, compensating for the loss of some pods and seeds due to high temperature [24,34,52].

In the case of the SEF 10 line (derived from the cross between *P. vulgaris* × *P. acutifolius* × *P. coccineus*), it has been characterized by CIAT’s bean breeding program as tolerant to terminal drought, high temperature, and acidic soil (low phosphorus and high aluminum), based on evaluations that were conducted at three contrasting field sites in Colombia, including Palmira (Valle del Cauca) for drought, Armero (Tolima) for high temperature, and Santander de Quilichao (Cauca) for acidic soil stress [48]. In our study, under the combined stress conditions of acidic soil and high temperatures, SEF 10 exhibited less abortion in the reproductive organs [24,53], as indicated by the production of buds and flowers. The SEF 10 vegetative and reproductive processes were not significantly affected by high temperatures, and the penalty observed in pod and grain formation was lower than that observed in BFS 10. SEF 10 responded to the increase in P supply with an increase in pod and grain production, thus allocating more energy to seed formation, so that seed viability was not drastically affected [54]. Increased P supply alleviated, to some extent, the effects of high temperature stress on reproductive organ development in both bean lines, but the effects seem to be a greater in regards to the BFS 10 compared to the SEF 10 line.

### 3.2. Phosphorus Supply Influenced Phenological Characteristics, Stomatal Density, and Dynamics of Morphological Development

The two bean lines evaluated appear to make adjustments that are related to the shortening of phenological stages (earliness or phenotypic plasticity) as a mechanism of adaptation to heat for achieving superior performance in terms of pod and grain production. Thus, the high temperature decreased the days to flowering (DF) and days to physiological maturity (DPM), this response being as part of an adaptation mechanism to cope with the incidence of heat stress [18,22,23,24]. When we analyzed differences in sink strength between treatments, we found a marked reduction with P0 treatment, as revealed by decreased values of PNA and SNA. The above may be due to the increased loss of pod and seed development (viability) caused by high daytime as well as nighttime temperatures [21,36], along with a very low supply of P. However, the earliness of both lines may not only be attributed to the high temperature escape response [22,50], but may also be due to the stimulatory effect of P on growth hormones that induce early flowering in common bean plants, as described by Bhattacharya [55], which translated into a strong correlation between phenological variables (DF and DPM) and yield [56]. 

Generally, pollen development and viability are affected by high temperatures [23,57]. In this study, we found that the PV values were lower (by about 60%) for the P0 treatment under the chronic high temperature stress conditions, in which the two bean lines faced high daytime (21.7 to 42.5 °C) as well as nighttime (23.9 ± 0.1 to 31 ± 0.2 °C) temperatures. Under these high temperature stress conditions, the two bean lines evaluated exhibited adjustments toward the higher production of flower buds and flowers as a compensation strategy for the reduced pollen viability. However, in treatments of increasing P supply, the effect of high temperature was reduced, a situation that resulted in higher values of pollen viability [21,50,58]. 

At the leaf level, heat stress was more severe in the P0 treatment, and this is due to adjustments made by the two bean lines in traits mainly related to leaf thickness, SLA, and stomatal density. These adjustments may be an attempt by the plant to maintain a higher water content in the leaf [59], as well as the better functioning of the photosynthetic apparatus [60]. But when the P supply is increased, the effect of high temperatures is notably reduced, as P is a vital component for the production of adenosine diphosphate (ADP) and adenosine triphosphate (ATP) [42,61], energy compounds that control most photosynthetic processes in bean plants [43,62]. Additionally, the bean lines maintained gas exchange characteristics with increased P supply, allowing the plant to function at the physiological level to sustain productivity [63]. This is due to the greater ability for ATP generation in the light reaction phase of photosynthesis, a situation that has been described in various studies [64,65]. This was also accompanied with changes in the morphological characteristics at the leaf level, resulting in larger and thinner leaves (higher values of SLA) that can intercept more light [60]. 

When analyzing the data for SD in both the leaf and the pod, it has been reported that SD levels depend on leaf expansion and the function of the stomata [66], with a lower density observed under an increasing P supply. However, higher SD values were found under PSOM treatment in which the P supply was not significantly high. It has been reported that the downregulation of stomatal development in the pods plays an important role in counteracting the effects of heat [67]. When we compared the two bean lines, the SD value was higher in BFS 10, but in general, there were no marked differences among treatments, i.e., the pod starts with a certain number of stomata, and these become more diffuse as the pod expands [66], with no significant changes in pod diameter among treatments. The influence of high temperature on SD values was similar for SEF 10 in all treatments. 

### 3.3. The Ability to Mobilize Photosynthates Contributed to Improved Agronomic Performance under Different Levels of P Supply as an Adaptive Trait to Cope with High Temperatures

Under the P0 treatment, the BFS 10 line adapted to high temperatures by the mobilization of assimilates from the canopy biomass (CB) toward pod formation [35,68]. However, with an increased P supply, BFS 10 adapted to heat stress through increased leaf and pod production. The BFS 10 line may have avoided heat stress with increased P supply through early pod maturation, combined with higher physiological efficiency in pod and seed filling [16]. A major adaptive strategy of BFS 10 under heat stress appears to be its greater capacity to mobilize assimilates from the pod wall to seed formation (PHI) [22,67]. Previous studies have indicated the importance of mobilizing photosynthates towards pod formation and seed production to achieve greater yield increases under stress conditions [69,70]. The results obtained in this study are consistent with previous reports.

When comparing the different P supply treatments, we found that the increased P supply allowed for a greater distribution of energy for the formation of different plant organs (flowers, leaves, pods, seeds), which varied between the two bean lines. These differences are mainly due to the increase in availability of P in the soil [52], as well as the capacity of the bean lines to use P efficiently [71]. This is because higher P nutrition under heat stress improves photosynthesis, water use efficiency, and grain size, which indicates the role of P in mitigating the adverse effects of high temperature [72,73]. Specifically, SEF 10 showed a greater (10% higher in SEF 10 compared to BFS 10) ability to allocate assimilates from vegetative structures toward pod (PPI) and seed (HI) production, as well as from the pod wall to the seed (PHI). However, BFS 10 managed to adapt to high temperature by compensating with a higher number of pods (PNA) and seeds (SNA), resulting in a higher GY at different levels of P supply.

### 3.4. Mechanisms of Energy Dissipation under Heat Stress Conditions under Different Levels of P Supply

We found that the two bean lines (BFS 10 and SEF 10) used different mechanisms to regulate energy, i.e., the balancing and/or performance adjustment mechanisms of PSI and PSII, the former being a protection of the photosynthetic apparatus from heat stress [74,75]. This is supported by the increase in the fraction of light dissipated as heat (NPQ). Thus, increasing the ambient temperature resulted in decreases in ΦPSII and photochemical quenching (qL and qP), which means that there was some level of inhibition of the electron transport chain (ETR) [76]. This inhibition of light-dependent reactions was accompanied by an increase in NPQ and qN [77,78], these adjustments being more striking under the P0 treatment in the two bean lines evaluated. These adaptive mechanisms enhance the dissipation of excess energy captured by the light-harvesting antenna to protect the photosynthetic apparatus [79] under low P. However, we found that ΦPSII is markedly reduced by low P, but it recovers after a subsequent period of adaptation, highlighting the highly reversible effect of low P (P0) on both lines [80]. Likewise, in the pod filling growth stage, the increase in F_o_ clearly indicated a lower energy capture efficiency of PSII; however, no inhibition of electron transport was observed, as the F_v_/F_m_ and ETR values were not compromised [45].

Similarly, the heat-induced reduction in Y(II) values was found to be mainly a consequence of increased non-photochemically regulated energy dissipation, as reflected in the change in Y(NPQ) and NPQ values [81], which increased mainly under P0 treatment in BFS 10 at the pod filling stage. This increase was a measure taken to protect PSII under excess irradiance, managing to reduce the excitation energy pressure on the PSII reaction centers [82,83], which could allow for the maintenance of the photosynthetic rates and the provision of a higher photoprotective capacity to avoid the photoinhibition of the plant to resist oxidative damage [84] by increasing the energy dissipation capacity, as detected by increases in non-photochemical quenching (measured as NPQ and qN), with no alterations in ΦPSII [76].

The qN was higher under increasing P supply treatments than that noted under P0 treatment, indicating a potential response to heat as the F_v_/F_m_ value increased, as well as a possible decrease in reactive oxygen species (ROS) accumulation to allow for the recovery from heat stress [81]. In addition, the enhanced responses of the bean lines with the increase in P supply possibly increased ATP synthase activity [85]. This increase may cause changes in the thylakoid membrane, as a photoprotective mechanism which occurs through the dissipation of excess excitation energy from light-harvesting complexes to prevent the overexcitation of PSII [86]. This could be an important sink mechanism for ATP and NADPH, thereby potentially decreasing the need for the thermal dissipation of energy and improving the photosynthetic response to heat stress [81]. 

Increasing P supply in the soil may dissipate, to some degree, the effects of high temperatures, as bean plants can, in addition to generating a physiological photoprotection mechanism (NPQ), make efficient use of energy oriented mainly to an increase in the electron transport rate (ETR) for photochemical processes (qP), as well as increase ATP production, which is necessary for CO_2_ fixation. Therefore, a P deficiency exacerbated high temperature stress in the two bean lines evaluated, with greater restrictions in growth and in the functioning of the photosynthetic apparatus. It has been reported that a lack of P in the soil can reduce plant growth, thus also reducing N demand and N_2_ fixation. Specifically, a low P supply affects the photosynthetic apparatus and therefore, the generation of non-structural carbohydrates to the nodules. Consequently, the activity of nitrogenase in the nodules is also affected [87], indicating that P is important for nodulation and symbiotic nitrogen fixation [88].

### 3.5. Increase in P Supply Could Act as a Heat Stress Dissipater under Climate Variability Scenarios

An increase in global mean surface air temperature of more than 1.5 °C is expected by the end of the 21st century [8], a situation that can significantly affect bean production. In addition, P is also a limiting factor in many regions of the world [33,89], and based on our results, P deficiency can increase the effect on beans of high temperatures resulting from climate change. Therefore, the tolerance of bean plants to heat stress is attributed to, among other factors, the greater acquisition of P from soil, as this allows the plant to avoid the disruption of the photosynthetic process while allowing the synthesis of mainly protective compounds [90]. Damage of any component of photosynthesis, such as the photosystems (PSI and PSII), the electron transport chain, or the CO_2_ reduction pathways, is sufficient to hinder the overall photosynthetic efficiency of a plant [91]. PSII is the most thermosensitive, which can lead to impairment of other cellular functions, such as the fluidity of the thylakoid membrane and the dependence of PSII on electron transport dynamics [92], after heat stress [81], as it can lead to the impaired coupling of ATP synthesis [93].

Evidence shows that under combined abiotic stress conditions, the physiological and agronomic responses are different compared to those noted under single stress [2,11,94]. The temperature and the availability of nutrients such as P are among the major drivers of plant productivity, and changes in these factors greatly influence plant adaptation to change in each environment [68]. Therefore, it is imperative that we develop crops with improved heat tolerance through plant acclimation [95]. In addition, alterations in plant growth and nutrient use response are expected due to interaction with environmental stress [96]. High temperatures tend to increase P concentration in plant tissues when soil P deficiency is present [97]. However, the effects of high temperature stress and its interactions with varying levels of P supply in the soil have rarely been evaluated [10,26,98] for developing a selection criteria for improving genetic adaptation. Thus, the evaluation of improved germplasm under combined stress conditions is necessary, particularly in the case of grain legumes, such as beans, in which P acquisition and utilization efficiency play an important role in plant growth, development, and yield stability in the face of climate change. 

## 4. Materials and Methods

### 4.1. Study Area and Experimental Setup

The evaluation of the bean lines was carried out at the Centro de Investigaciones Amazónicas CIMAZ Macagual of the Universidad de la Amazonia (1°37′ N 75°36′ W), located in Florencia, Caquetá (Colombia) in a screenhouse over two seasons: (i) October 2019 to January 2020; and (ii) September to December 2020. The environmental conditions during the experiment outside (ambient or field conditions) varied with respect to those inside of the screenhouse (Figure 1). The average outside minimum and maximum daytime temperature was 22.3 °C and 32.2 °C, respectively, while the average outside nighttime temperature was 22.4 °C and 24.9 °C, respectively. During the growing period inside the screenhouse, the mean minimum and maximum daytime temperatures were 24.1 °C and 37.9 °C, respectively, while the mean minimum and maximum nighttime temperatures were 23.9 °C and 27.3 °C, respectively, with mean relative humidity levels of 66.6% during the day and 86.5% at night (Supplementary Figure S1). The temperature conditions inside the screenhouse were considered to be significantly above the thresholds at which beans experience heat stress under field conditions during the day and particularly, at night [22,23,24,58].

The two interspecific Mesoamerican lines (BFS 10 and SEF 10) selected for the study were of the indeterminate growth habit type II. The BFS 10 line was developed from a cross of [(SER 76 × RCB 589)F_1_ × (SXB 407 × SER 119)], while the interspecific SEF 10 line originated from a cross of [(ALB 74 × INB 841)F_1_ × RCB 593], where ALB 74 contributed genes from *P. coccineus*, INB 841 from *P. acutifolius*, and RCB 593 from *P. vulgaris*. These lines were selected from breeding programs based on their ability to adapt to acidic soils with low fertility and under high temperatures [21,22,23,99], as well as their resistance to different biotic stress conditions [21,22,23,99]. The BFS (small red) line is better adapted to low soil fertility, while the SEF (red) line is improved for adaptation to both drought and heat. A randomized complete block (RCB) design was used under a factorial arrangement (five levels of P × 2 genotypes) for a total of 10 treatments with four replications. The factors considered in the experiment were: (i) five P supply levels (kg ha^−1^): four levels of P0, P15, P30, and P45, through application of rock phosphate (RP), and one P supply level achieved through the application organic matter (PSOM) corresponding to 25 kg P ha^−1^ (P25); and (ii) two advanced bean lines (BFS 10 and SEF10). The soil used for the evaluation is a clay loam oxisol characterized by low pH 4.22, with high aluminum (Al) saturation (73.4%), exchangeable Al content (6.3 cmol(+) kg^−1^), and exchangeable soil acidity (1.76 cmol kg^−1^). The soil organic carbon (SOC) content was 3.58%, while the available P content was 1.04 mg kg^−1^ (Bray-II). The cation exchange capacity (CEC) of the soil was 17.5 cmol kg^−1^, with a total base saturation of 3.92% (cmol kg^−1^: Ca: 0.25, Mg: 0.11, K: 0.09, Na: 0.24). 

Each soil block was a raised bed measuring 1.5 m wide by 8 m long, with a depth of 40 cm and a separation between beds of 1.2 m, and the experiment comprised five treatments. In each experimental unit, corresponding to the treatment, four 1.5 m long rows were planted, with a distance between rows of 0.4 m and a distance between plants of 10 cm (equivalent to 15 plants m^−2^). The amount of P supply (through P application) used in the study was based on those recommended for tropical soils, representing (kg ha^−1^) P0, P15, and P45 as deficient, low, and high P supply, respectively, for leguminous crops such as beans. An additional included P treatment consisted of P supplied through organic matter (PSOM), achieved through the application of organic matter (OM) at 2.5 ton ha^−1^ corresponding to 25 kg P ha^−1^. This P treatment is equivalent to a smallholder nutrient management option under local conditions. 

During the experiment, plants were watered daily by drip irrigation at field capacity to eliminate the effect of water limitation on reproductive development. Soil moisture content was monitored using an EC-5 (Decagon devices, Inc) soil moisture sensor. The inorganic source used for P supply was the application of rock phosphate (RP) “Phosphorite, INFERHUILA S.A” [100]. This is a suitable P source for legume production in acidic soils. The RP used presents a composition of 3% assimilable P (P_2_O_5_), 21% slow assimilation P (P_2_O_5_), and 32% Ca (CaO); a total of RP (kg ha^−1^) 500, 1000, and 1500, equivalent to assimilable phosphorus (kg ha^−1^) P15, P30, and P45, were applied; all P treatments were treated with organic matter (2.5 ton ha^−1^) to improve RF efficiency in regards to P availability in acidic soils, and all treatments were established 15 days before planting. No other fertilizers were applied, and the plants were protected from pests and diseases. The application of RP and OM significantly increased the availability of P in the soil, reduced soil acidity, and improved the availability of nutrients needed for the growth of the bean crop.

### 4.2. Dynamics of Growth and Development of Different Vegetative Organs

To determine the effect of P supply on the dynamics of growth and development of the different vegetative organs in two bean lines grown under high temperature conditions in acidic soil, plant samples were collected at 14 time points from 10 to 62 days after planting (DAP). In each sampling time, four plants (n = 80 plants monitored for each sampling period, with a total of 1120 plants monitored during the experiment) were randomly selected per treatment, and the following measurements were obtained: plant height (cm); stem diameter (mm); and the number of leaves, flower buds, flowers, and pods. Also, at physiological maturity, destructive sampling (n = 10 plants per treatment, for a total of 200 plants per monitoring period) was carried out to determine the effect of P supply on pod formation in terms of the number and weight of the pods per plant, under high temperatures and acidic soil stress conditions, as well as the number of developed (viable) and undeveloped (non-viable) seeds per pod and the total number of seeds per plant, the total weight of viable seeds, and the average weight per seed. 

### 4.3. Biomass Partitioning and Grain Yield

To evaluate differences in grain yield, destructive sampling was conducted in the central part of each plot. The pods were threshed from harvested plants and the grains were cleaned and dried to determine the grain yield (kg ha^−1^). The seeds collected at harvest time were homogenized, and 100 seeds were randomly selected to determine their weight (g). In each plot, a 0.5 m row segment (equivalent to an area of 0.3 m^2^) was selected, five plants were collected for destructive sampling, and this activity was carried out during the mid-pod filling phase, which corresponds to the time when 50% of the pods have reached their final length, i.e., approximately 55 days after sowing, according to the BBCH scale for bean growth (BBCH 75). Another destructive sampling was also carried out at harvest time (BBCH 89), when the dry biomass of the different plant components (leaves, stems, pods, and seeds) was recorded, as well as the seed number per area (SNA) and the pod number per area (PNA). Dry matter partitioning indices, such as the pod partition index (PPI), the pod harvest index (PHI), and the harvest index (HI) were determined as described previously [99].

### 4.4. Phenological Characteristics, Stomatal Density, and Morphological Development

To determine the influence of P supply on the phenological traits of two bean lines grown under acidic soil and high temperature stress conditions, the number of days to flowering (DF) and days to physiological maturity (DPM) were measured, as well as the pollen viability (PV), as described by Suárez et al. [22,23,24]. Likewise, as an adaptive variable, the specific leaf area (SLA) was calculated using the methodology described by Cornelissen et al. [101]. For SLA, a total of 1080 leaf discs were used, corresponding to 6 discs per leaf, 9 leaves per treatment (P supply), and 10 treatments at two phenological phases of flowering (R_6_) and mid-pod filling (R_8_). The MultispeQ handheld device [102] was used for measuring leaf thickness. 

Stomatal density (SD, the number of stomata per unit area) was determined on samples taken from the fourth fully expanded leaf (BBCH 65), a process that was also carried out on mature pods (BBCH 75). Nail polish was applied to each organ and allowed to dry for about 5 min; then, the glaze layer was carefully removed with a tape and adhered to a slide. This procedure was performed on four leaves and pods of each treatment, for a total of 160 units that were monitored for both leaves and pods. In the leaves, stomatal impressions were made on the adaxial surface of the leaf, in the area between the mid-vein and the margins. In the case of pods, stomatal impressions were made on both sides of the mid-section of the pod. For each slide, two random counts were conducted to determine the density of the stomata (number mm^−2^). 

Regarding the dynamics of morphological development, the growth rate for both the length and width of the leaflets was measured, an activity that was carried out with an interval of five days at each sampling for a total of six samplings for each phenological stage. A total of 360 leaves (1080 leaflets) were used, corresponding to 12 leaves per treatment, and 10 treatments in three phenological stages (first leaf V_3_, pre-flowering R_5_, and flowering R_6_). Likewise, the speed of pod growth was determined by measuring the length and width of the pods with a five-day interval for each sampling. This activity was carried out during five samplings with different groups of pods that were developed at 36 and 41 days after planting (DAP). The pod length (mm) was measured from the base to the apical end, and the pod width (mm) was measured from the ventral suture to the ventral dorsum of the pod. For each developmental period, 12 pods were used (36 and 41 DAP) for a total of 24 pods per each treatment (n = 480 pods corresponding to 24 per treatment for five levels of P supply and four replications (blocks) in each period of the experiment). 

### 4.5. Chl_a_ Fluorescence and Imaging for the Chlorophyll (Chl_a_) Fluorescence Parameters under Different Levels of P Supply

The Chl_a_ fluorescence images and parameters were determined using the Imaging-PAM M-series fluorometer and Imaging WIN version 2.32 software (Heinz Walz GmbH, Effeltrich, Germany). Measurements were performed on the fourth fully developed trifoliate leaf (counted from apex to base) at two phenological stages, flowering (BBCH 65) and mid-filling (BBCH 75). The leaves were initially dark adapted for 30 min. The leaves were then exposed to a standard light curve in 12 illumination steps (ranging from 0 to 701 µmol m^−2^ s^−1^ PAR) (time/10 s between steps). Initially, upon induction of this curve, the equipment produced a weak measurement beam (0.5 µmol m^−2^ s^−1^, 100 µs, 1 Hz) to determine the initial fluorescence (F_0_) open reaction centers of PSII. Next, a saturating white light pulse of 2400 µmol m^−2^ s^−1^ (10 Hz) was emitted for 0.8 s to reach the maximum fluorescence emission (F_m_) of the closed PSII reaction centers. From these measurements, the maximum photochemical efficiency of PSII (F_v_/F_m_) [F_v_/F_m_ = (F_m_ − F_0_)/F_m_] was estimated [103,104]. The leaves were then exposed to actinic photon irradiance (110 µmol m^−2^ s^−1^) for 120 s to obtain the steady-state fluorescence yield (F_s_), then a saturating light pulse of 2400 m^−2^ s^−1^ was applied for 0.8 s to reach the maximum light-adapted fluorescence (F_m_′). The initial light-adapted fluorescence (F_0_′) was estimated according to the methods of Oxborough and Baker [105]. Following the calculations proposed by Kramer et al. [106], the energy adsorbed by PSII was determined for the two yield components for the dissipative processes: the effective quantum yield of PSII Y(II) [Y(II) = (F_m_′ − F)/F_m_′], and the yield of dissipation by deregulation Y(NPQ) [Y(NPQ) = (F_s_/F_m_′) − (F_s_/F_m_)], as well as the apparent electron transport rate (ETR) [ETR = 0.5 × Y(II) × PAR × absorptivity], the photochemical quenching coefficient qP [qP = (F_m_′ − F)/(F_m_′ − F_o_′)], the non-photochemical quenching coefficient qN [qN = (F_m_ − F_m_′)/(F_m_ − F_o_′)], the yield for dissipation by downregulation [NPQ = (F_s_/F_m_′) − (F_s_/F_m_)], and the yield for other non-photochemical (non-regulated) losses [NO = F_s_/F_m_]. These variables were measured on fully developed leaves at flowering and pod filling, following the methodology described by Rios et al. [107] to capture Chl_a_ fluorescence emission transients.

### 4.6. Data Analysis

A linear mixed model (LMM) was fitted to analyze the effect of fixed factors (five P supply levels and two advanced bean lines). Random effects were included as nested blocks within the growth phase of the evaluation in each season. Assumptions of normality and homogeneity of variance were assessed by exploratory analysis of the residuals. Differences between treatments were analyzed with Fisher’s post-hoc LSD test, with a significance of α = 0.05. Box plots were prepared for the measured variables using the results from the analysis of variance. A Pearson correlation analysis was also performed to determine the relationship between grain yield and the other agronomic and physiological variables of each bean line under each level of P supply. Also, to visualize the correlations in a general way, a chord diagram was developed using the corrplot [108] and circlize [109] packages. The LMMs were performed using the lme function of the nlme package, and the graphical outputs were performed in the “ade4”, “ggplot2”, “factoextra” and “corrplot” packages in the R language software, version 4.2.0 [110].

## 5. Conclusions

Increasing the supply of phosphorus (P) under high temperature stress caused a significant increase in the growth and development dynamics of different vegetative organs, as well as in the efficiency of photosynthesis and photosynthate remobilization, and these adaptive responses resulted in higher grain yields for the two bean lines (BFS 10 and SEF10) evaluated. Both bean lines showed adaptive mechanisms related to phenological adjustments and heat dissipation. Thus, the increased P supply may have alleviated the effects of high temperature stress on the growth and development of the reproductive organs. The results from this study show that the BFS 10 and SEF 10 lines may serve as useful parents in a bean breeding program to combine the adaptation to multiple abiotic stress factors.

## Data Availability

Data are available from the authors upon request.

## Supplementary Materials

The following supporting information can be downloaded at: https://www.mdpi.com/article/10.3390/plants12183277/s1, Figure S1. Distribution of maximum/minimum day and night temperatures and relative humidity during the growing season at the Macagual Research Center in Colombia over two seasons (2019) and (2020). (a) Temperature—the dotted horizontal line denotes 22 °C and 32 °C; (b) relative humidity (%)—the dotted horizontal line denotes 60% relative humidity. Green dashed vertical lines on the X axis indicate days to flowering and days to physiological maturity. Figure S2. Response of two bean lines (BFS 10, SEF 10) to different levels of P supply (P0, P15, P30, and P45 kg ha^−1^; and P supplied through organic matter (PSOM)) to acidic soil under high temperature stress conditions. (a) Days to flowering (DF); (b) days to physiological maturity (DPM); (c) pollen viability (PV); (d) stomatal density (SD). Figure S3. Leaf growth response of two bean lines (BFS 10, SEF 10) to different levels of P supply (P0, P15, P30, and P45 kg ha^−1^; and P supplied through organic matter (PSOM)) to acidic soil under high temperature stress conditions. (a) Specific leaf area (SLA); (b) leaf thickness. Figure S4. Leaflet growth rate response of two bean lines (BFS 10, SEF 10) to different levels of P supply (P0, P15, P30, and P45 kg ha^−1^; and P supplied through organic matter (PSOM)) to acidic soil under high temperature conditions. (a) Length; (b) width. Figure S5. Pod growth response of two bean lines (BFS 10, SEF 10) to different levels of P supply (P0, P15, P30, and P45 kg ha^−1^; and P supplied through organic matter (PSOM)) to acidic soil under high temperature stress conditions. (a) Length; (b) width.
