# Supplementary figures and images for: Influence of Increase in Phosphorus Supply on Agronomic, Phenological, and Physiological Performance of Two Common Bean Breeding Lines Grown in Acidic Soil under High Temperature Stress Conditions
Source: Plants (Basel). 2023 Sep 15;12(18):3277. doi: 10.3390/plants12183277 (PMC10534644; doi:10.3390/plants12183277)

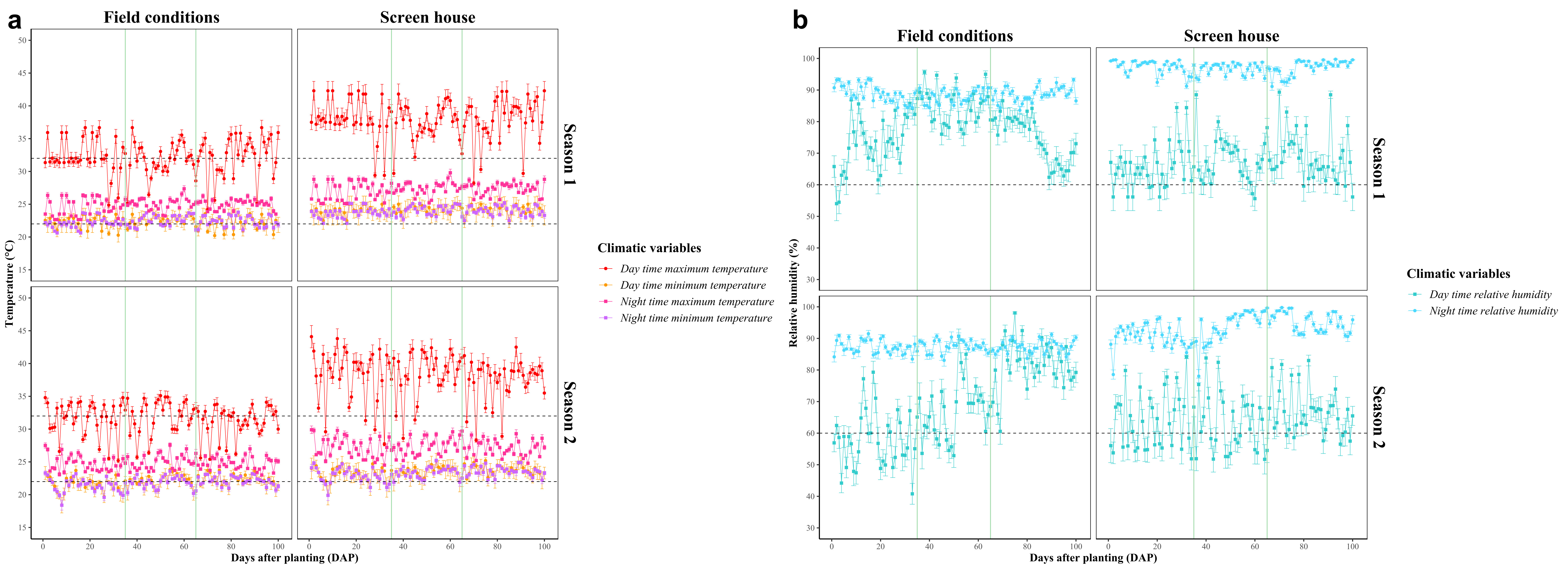

Supplement: Supplementary file 1 [file plants-12-03277-s001.zip › Supplementary Figure S1.jpg]

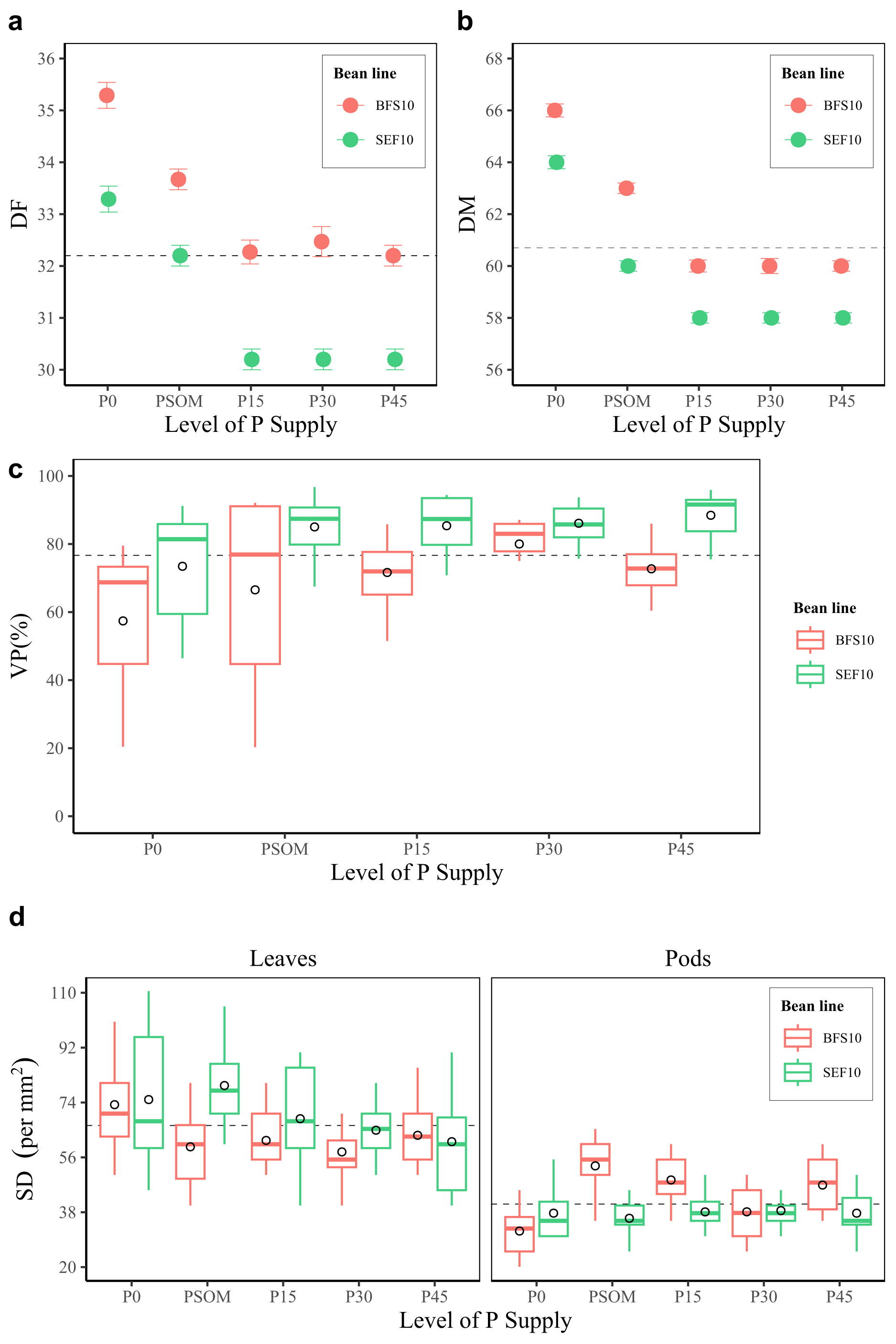

Supplement: Supplementary file 1 [file plants-12-03277-s001.zip › Supplementary Figure S2.jpg]

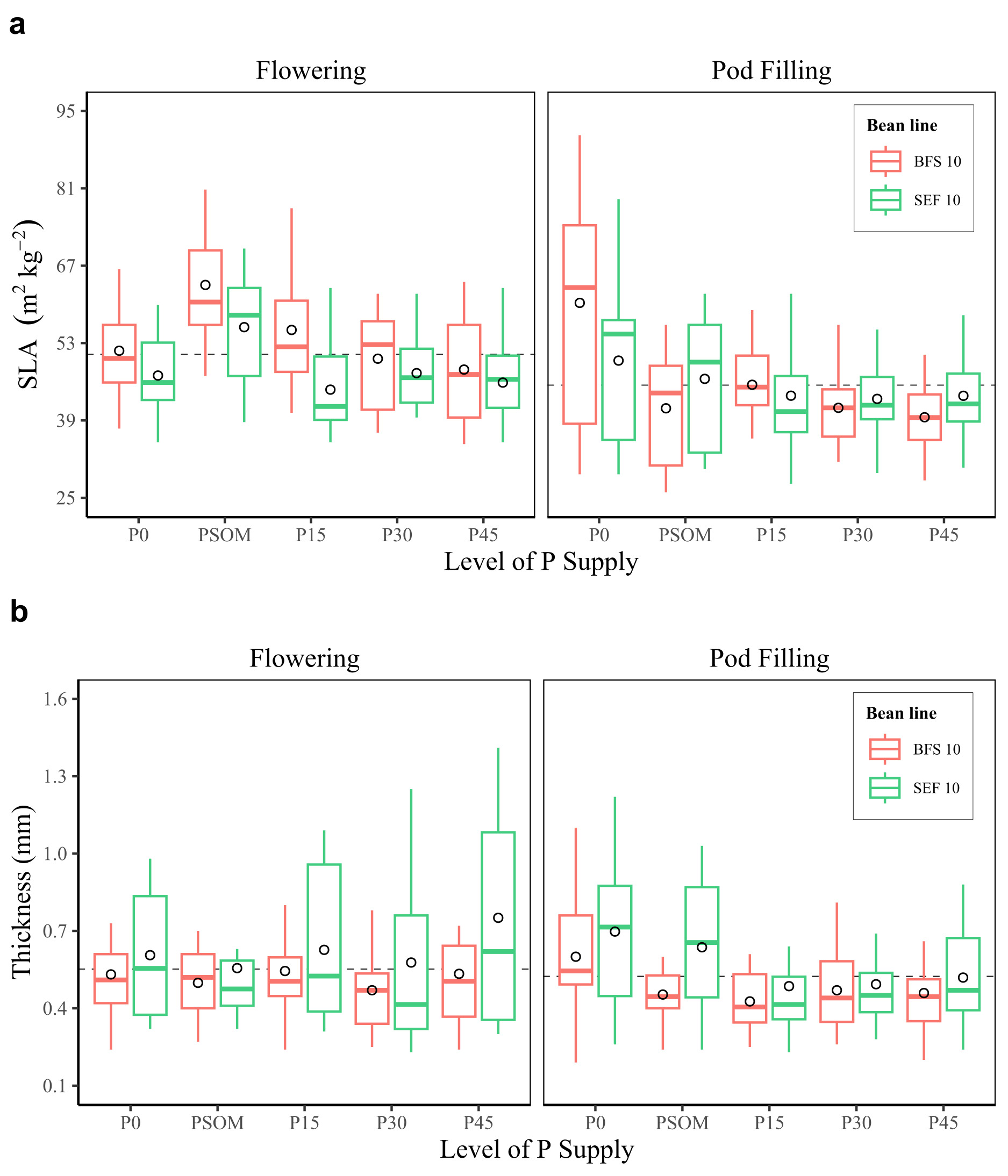

Supplement: Supplementary file 1 [file plants-12-03277-s001.zip › Supplementary Figure S3.jpg]

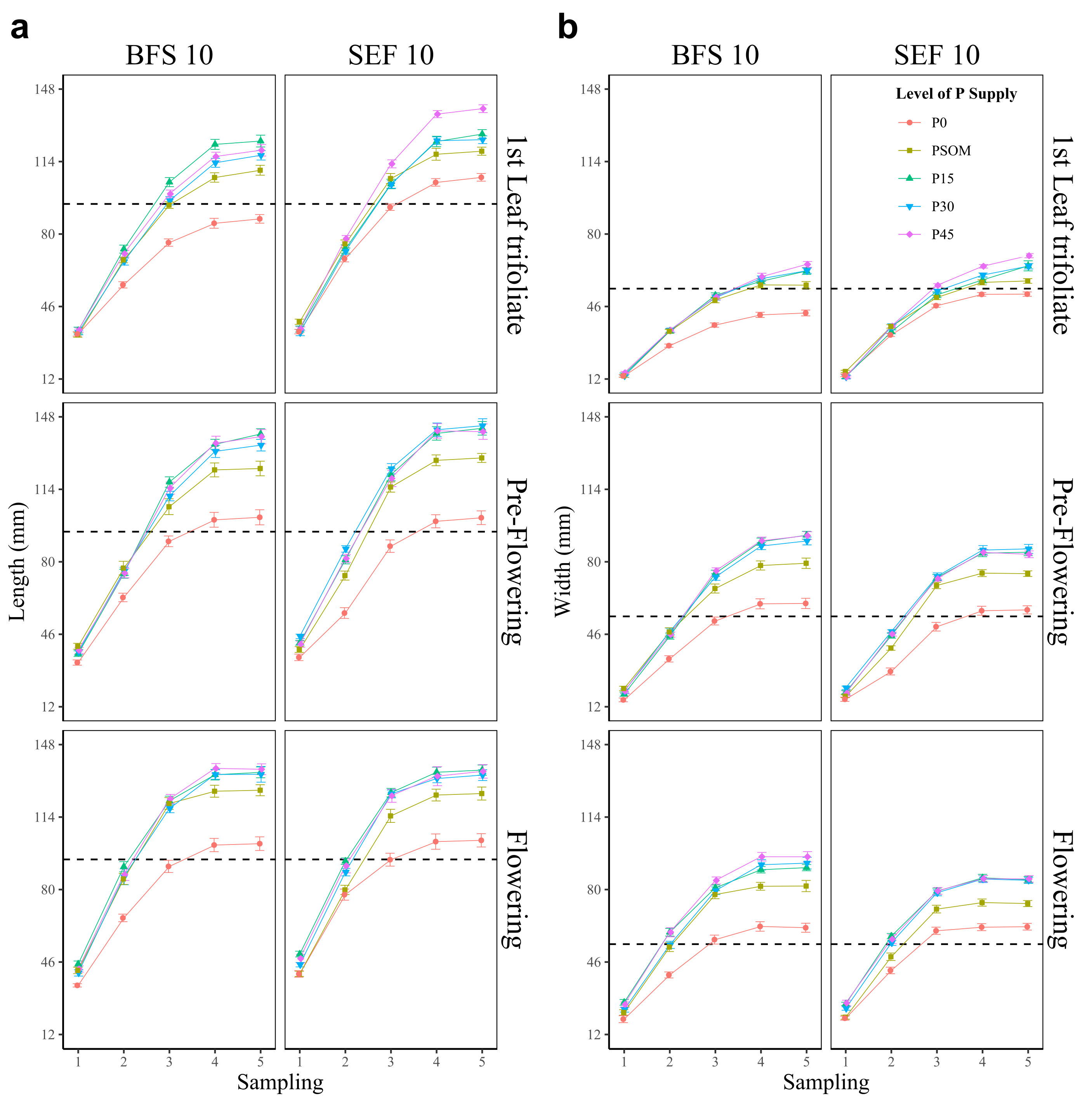

Supplement: Supplementary file 1 [file plants-12-03277-s001.zip › Supplementary Figure S4.jpg]

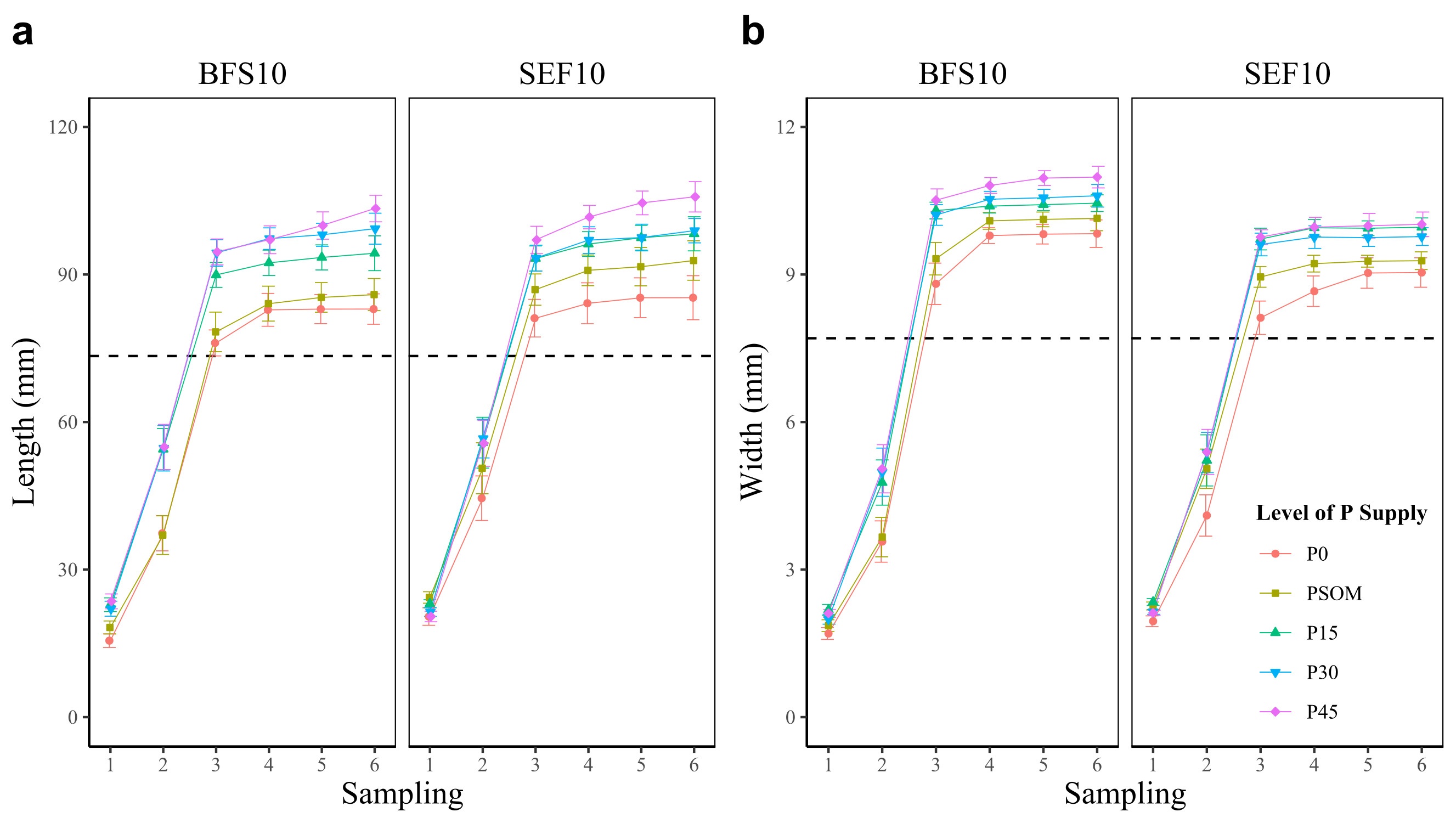

Supplement: Supplementary file 1 [file plants-12-03277-s001.zip › Supplementary Figure S5.jpg]
